# The non-canonical Notch signaling is essential for the control of fertility in *Aedes aegypti*

**DOI:** 10.1371/journal.pntd.0006307

**Published:** 2018-03-05

**Authors:** Chia-Hao Chang, Yu-Ting Liu, Shih-Che Weng, I-Yi Chen, Po-Nien Tsao, Shin-Hong Shiao

**Affiliations:** 1 Department of Parasitology, National Taiwan University, Taipei, Taiwan; 2 Department of Pediatrics, National Taiwan University Hospital, Taipei, Taiwan; 3 Research Center for Developmental Biology & Regeneration Medicine, National Taiwan University, Taipei, Taiwan; University of Wisconsin Madison, UNITED STATES

## Abstract

The Notch signaling pathway is a highly evolutionarily-conserved cell-cell signaling pathway that regulates many events during development. It plays a pivotal role in the regulation of fundamental cellular processes, such as cell proliferation, stem cell maintenance, and differentiation during embryonic and adult development. However, functions of Notch signaling in *Aedes aegypti*, the major mosquito vector for dengue, are largely unknown. In this study, we identified a unique feature of *A*. *aegypti* Notch (AaNotch) in the control of the sterile-like phenotype in female mosquitoes. Silencing AaNotch with a reverse genetic approach significantly reduced the fecundity and fertility of the mosquito. Silencing AaNotch also resulted in the prevention of micropyle formation, which led to impaired fertilization. In addition, JNK phosphorylation (a signaling molecule in the non-canonical Notch signaling pathway) was inhibited in the absence of AaNotch. Furthermore, treatment with a JNK inhibitor in the mosquito resulted in impaired fecundity and fertility. Taken together, our results demonstrate that non-canonical Notch signaling is essential for controlling fertility in the *A*. *aegypti* mosquito.

## Author summary

Mosquitoes transmit many devastating diseases, including malaria, dengue, and Zika, which together are responsible for over one million deaths per year. Major reasons for this tragic situation are the unavailability of effective vaccines and drugs for most mosquito-borne diseases, increased resistance of vectors to insecticides, and resistance of pathogens to currently available drugs. A thorough understanding of the molecular machinery involved in mosquito fertility is essential for developing vector control strategies. In this study, we observed a unique feature of the *Aedes aegypti* Notch (AaNotch) in the control of a sterile-like phenotype in female mosquitoes. Silencing AaNotch using a reverse genetic approach revealed significant reductions in fecundity and fertility. It also resulted in the abolishment of micropyles, which led to impaired fertilization. However, no effect on fecundity and fertility was observed in the absence of AaDelta, a canonical Notch transmembrane ligand. Although JNK is a downstream component of the non-canonical Notch signaling pathway, treatment with a JNK inhibitor resulted in impaired fecundity and fertility. In conclusion, our results demonstrate that Notch-dependent regulation of sterile-like female mosquitoes is controlled by non-canonical Notch signaling.

## Introduction

Mosquitoes are highly-effective vectors that transmit many devastating diseases, including malaria, dengue, and Zika. Together, these diseases are responsible for over one million deaths each year [1–4]. Of note, cases of dengue are reaching disastrous levels in Central and South America and in Southeast Asia [5–7]. Recently, the outbreak of Zika became a threat to global health and now poses a significant public health challenge [8, 9]. Major reasons for this tragic situation are the unavailability of effective vaccines, an increase of vector resistance to insecticides, and pathogen resistance to drugs [10–12].

Most mosquitoes can obtain amino acids and other nutrients needed for egg development from the blood of their vertebrate hosts. A blood meal results in a highly-regulated cyclicity in egg production, with each cycle tightly coupled to blood intake [13]. Mosquito vitellogenesis is initiated following a blood meal. A blood meal induces the production of ovarian ecdysteroidogenic hormone from the mosquito’s brain, which stimulates the production of ecdysone in follicle cells [14]. Ecdysone is then converted to 20-hydroxyecdysone (20E) to activate the production of yolk protein precursors in fat bodies [13]. The target of rapamycin (TOR) signaling pathway has been shown to serve as a key cell regulator needed to complete vitellogenesis [15, 16]. TOR signaling is regulated by rapidly increasing concentrations of specific amino acids in the hemolymph post blood meal (PBM) particularly leucine [17]. Inhibition of TOR in fat body culture systems, by either rapamycin or RNA interference (RNAi)-mediated gene depletion, results in a significant down-regulation of Vg gene transcription after amino acid stimulation [15, 16]. In addition, inhibition of TOR *in vivo* inhibits egg development [15, 16]. Results of these studies suggest that a thorough understanding of the molecular machinery involved in mosquito fertility will be useful for developing vector control strategies.

The Notch gene was discovered by Morgan et al, who observed that a partial loss of Notch function results in the formation of notches at the wing margins of *Drosophila melanogaster* [18]. Experiments in the early 1980s established that the Notch gene encodes a 300-kDa, single-pass transmembrane receptor. In addition, the extracellular domain of the Notch receptor contains 36 epidermal growth factor (EGF)-like repeats that are essential for ligand binding, whereas the intracellular domain is involved in cellular signaling and contains multiple conserved protein domains. Notch-like molecules have been identified in a wide-range of organisms, from free-living nematodes (e.g., *Caenorhabditis elegans*) to humans, suggesting that they have important (and apparently conserved) functional roles in embryonic development [19]. However, it is not clear what role Notch signaling plays in the development of specific tissues or how it might activate downstream genes [20].

The most extensively characterized signaling pathway is known as the canonical Notch signaling pathway [21]. In canonical Notch signaling, a Notch transmembrane receptor interacts with a ligand (Delta) on a neighboring cell, followed by a proteolytic cleavage of the receptor and the subsequent release of the Notch intracellular domain (NICD). Translocation of NICD to the nucleus leads to its interaction with a CBF1/Suppressor of the Hairless/LAG-1 (CSL) family DNA-binding protein, which results in the transcription of Notch target genes [22]. In contrast, non-canonical Notch signaling has been shown to differ markedly from canonical Notch signaling in that the initiation of non-canonical Notch signaling may function without ligand binding [23, 24]. The JNK pathway is activated when a MAPK kinase (Hemipterous or Hep in *Drosophila*) phosphorylates JNK, which in turn, phosphorylates the downstream AP-1 transcription factors Jun and Fos. Notably, JNK has been implicated as an important factor in egg-micropyle development in fruit flies and participates in the CSL-independent, non-canonical Notch signaling pathway [25, 26].

In this study, we observed a unique feature of *Aedes aegypti* Notch (AaNotch) in the control of a sterile-like phenotype in female mosquitoes. Silencing AaNotch in the mosquito (but not AaDelta, a canonical, Notch transmembrane ligand) using a reverse genetic approach resulted in a significant reduction in fecundity and fertility. Silencing AaNotch abolishes micropyles, which leads to impaired fertilization. Although JNK is a downstream molecule of the non-canonical Notch signaling pathway, chemical inhibition of JNK results in impaired fecundity and fertility. Taken together, our results demonstrate that Notch-dependent regulation of sterile-like female mosquitoes is controlled by non-canonical Notch signaling.

## Methods

### Mosquitoes

The *A*. *aegypti* UGAL/Rockefeller mosquito strain used in this study and was raised (with slight modification) in manner described by other investigators [27, 28]. Briefly, mosquitoes were provided with 10% sucrose solution and maintained at 28 °C in 75%–80% humidity with a 12/12 h light/dark cycle. Both males and females were kept in the same cage until a blood meal was provided to the females. Female mosquitoes at 3–5 days post eclosion were allowed to feed on anesthetized ICR mice (Institute of Cancer Research, USA) to initiate egg development. The ICR mice used in this study were obtained from the Laboratory of Animal Center at National Taiwan University (Taipei, Taiwan). Females that had not obtained blood from the mice were immediately separated from those that had. Only blood-fed females were used for further experiments and egg-laying behavior was observed between 72 and 96 h PBM.

### Ethics statement

The research plan for animal use was approved by the Laboratory of Animal Center at National Taiwan University (Taipei, Taiwan) under approval ID #20100268. All procedures and care are described in the Standard Operating Procedure of the Laboratory of Animal Center at National Taiwan University. All persons involved in animal work had successfully completed Animal Care Training at National Taiwan University (Taipei, Taiwan) and were specifically trained in protocols used in the research plan.

### Cloning and sequencing of Notch cDNA

Standard procedures were used in recombinant DNA manipulations. Expressed sequence tag cDNA sequences coding for the *Notch* gene were identified in the VectorBase database (https://www.vectorbase.org), using *Drosophila* Notch protein as the template (tBLASTn). Full-length Notch cDNA from the cDNA pool of *A*. *aegypti* was amplified with PCR using gene-specific primers. All PCR products were cloned into the pCRII-TOPO vector (ABI/Invitrogen, Carlsbad, California, USA). Full-length cDNA, deduced amino acid sequences and sequence alignment of *Notch* were compared using the BLAST tool provided by the National Center for Biotechnology Information (https://blast.ncbi.nlm.nih.gov/Blast.cgi?PAGE_TYPE=BlastSearch&BLAST_SPEC=blast2seq&LINK_LOC=align2seq) via the Clustal algorithm.

### RNA extraction, reverse-transcription, and quantitative real-time PCR

Total RNA from dissected mosquitoes was extracted with TRIzol (ABI/Invitrogen, Carlsbad, California, USA) and reversely transcribed. Quantitative PCR (qPCR) was performed using the ABI 7900 system (ABI/Invitrogen, Carlsbad, California, USA) and reactions were performed in 96-well plates using specific primers for AaNotch and the S7 ribosomal protein gene (internal control). ABI supermix (ABI/Invitrogen, Carlsbad, California, USA) was used for the SYBR green reaction. All qPCR reactions were run in duplicate using 2 μl cDNA per reaction. For each experiment, data were generated from at least three different cohorts of female mosquitoes. Quantitative measurements were performed in triplicate and normalized against S7 ribosomal mRNA. A fold-change value was derived using the 2^-ΔΔ^Ct method. Time points chosen to characterize the complete vitellogenic cycle were: pre-vitellogenesis (3–4 days post eclosion), vitellogenesis (6, 12 and 24 h PBM), early post-vitellogenesis (48 h PBM), and late post-vitellogenesis (72 h PBM). Standard curves for qPCR experiments were generated using a serial dilution of plasmids containing the transcript of the gene of interest [27]. The lowest dilution of the standard curve was given an arbitrary value of 10^5^ and so subsequent values for serial dilutions were five orders of magnitude lower. Amounts of amplicon in test samples were generated by comparing them with the standard curve. Hence, the term “relative” means that the samples were measured relative to the standard curve of the gene of concern. The number on the *Y*-axis thus represents a relative value and so has no unit. Primers were as follows: S7 forward (5′-TCAGTGTACAAGAAGCTGACCGGA), S7 reverse (5′-TTCCGCGCGCGC-TCACT-TATTAGATT), AaNotch qPCR-F (5′-GCGTTTCGGTGCTGCTTAG), AaNotch qPCR-R (5′-CCAATTGCTGGAATCTGTTACG), AaNotch RNAi-F (5′-TAATACGACTCACTATAGGGCTCAATGGGGCAGAGTTCAT), and AaNotch RNAi-R (5′-TAATACGACTCACTATAGGGCTACCGTTTTGCCAGACCAT). Primers used specifically for reverse-transcription PCR analysis were AaNotch forward (5′-ACTGTG-CGAACGCAATTCGGAAGC) for RNAi confirmation and AaNotch reverse (5′-GGCTACTG-TGATTGGGCTGGGGAGA) for RNAi confirmation. All other primers used in this study are listed in S1 Table. Amplifications were performed with SYBR Green PCR master mix (ABI/Invitrogen, Carlsbad, California, USA) and analyzed using the ABI PRISM 7900 sequence detection system (following the manufacturer’s instruction). Raw data were exported to EXCEL (Microsoft) for analysis.

### RNA interference

To generate double-stranded RNA (dsRNA) female mosquitoes were injected with 1 μg of AaNotch dsRNA (3μg/μL) using a Nanoject II injector (Drummond, Broomall, Pennsylvania, USA) following procedures described previously [28]. After four days of recovery, mosquitoes were given a blood meal and examined for AaNotch depletion. Control LacZ dsRNA containing a nonfunctional part of the *E*. *coli* LacZ gene was amplified from the DH5α strain.

### Scanning electron microscopy

Mosquito eggs were pre-fixed with 4% glutaraldehyde for 1 h and then post-fixed in 1% osmium tetroxide for 1 h. Each sample was washed three times with 0.1 M phosphate buffer (pH 7.4). Then, samples were dehydrated for 30 min each with increasing concentrations of ethanol (30%, 50%, 70%, 90%, and 100%) and then placed in 100% acetone for another 30 min. Subsequent critical-point drying and gold coating of particles were performed by the National Taiwan University TechComm. Gold-coated egg samples were analyzed with an FEI Inspect S scanning electron microscope (Thermo Fisher Scientific, Inc.).

### Hatching assay

Female mosquitoes at 3–5 days post-emergence were given a blood meal. Then, three days after obtaining a blood meal, mosquitoes were placed individually into a 50 mL centrifuge tube with a wet piece of 3M paper on which they could oviposit. The 3M papers were then dried and kept at room temperature for at least five days. Then, the 3M papers were placed in 20–30 °C water and exposed to a vacuous atmosphere for 1 h for hatching. Numbers of hatched larvae were calculated to compare hatching rates.

### Protein extraction and western blot analysis

Mosquito tissues collected from individual mosquitoes were separately put into micro-centrifuge tubes containing 100μL of breaking buffer [50 mM Tris (pH 7.4), 1% IGEPAL, 0.25% sodium deoxycholate, 150 mM NaCl, 1 mM EDTA, 1 mM phenylmethyl-sulfonylfluoride, 1X protease inhibitor mixture, and 1X phosphatase inhibitor mixture (Sigma-Aldrich, St. Louis, Missouri, USA)] and homogenized using a pellet pestle. The homogenates were centrifuged at 13,000 rpm for 5 min. The supernatants were transferred into a Qiashredder Column (Qiagen, Los Angeles, California, USA) and centrifuged again under the same conditions for 10 min. The flow-through was transferred to a clean micro-centrifuge tube to conduct a Western blot analysis using anti-phosphoric JNK antibody (V7931, Promega) and anti-JNK antibody (sc-571, Santa Cruz). The blot was developed by VisGlow Chemiluminescent Substrate and HRP (Visual Protein).

### Treatment with JNK inhibitor (SP600125)

Female mosquitoes at three to five days post-emergence were fed with blood they obtained from anesthetized ICR mice. Each mosquito was injected with 0.75 μg of SP600125 at 24 h PBM. Mosquitoes injected with dimethyl sulfoxide (DMSO) were used as controls.

### Statistical analyses

All statistical analyzes in this study were performed using GraphPad Prism 5 software (GraphPad Prism software). Gene-expression, fecundity, and fertility data were analyzed using ANOVA for all independent experiments.

## Results

### Cloning and characterization of Notch in *A*. *aegypti*

We cloned Notch cDNA from the mosquito *A*. *aegypti* UGAL/Rockefeller strain (Vector Base ID: AAEL001210). The cDNA encodes for a deduced 2599 amino acids with a relative molecular mass of approximately 285.8 kDa. To decipher the specific expression patterns of AaNotch in various tissues, we examined the transcription level of AaNotch in fat bodies (homologous to the mammalian liver), midgut, ovary, and carcass (a collection of the remaining tissues). Analysis of three independent cohorts showed that AaNotch transcripts were expressed in ovary, midgut, and fat body in response to a blood meal. It is worth noting that AaNotch transcription was greatly increased in ovaries PBM (S1 Fig).

### Silencing AaNotch inhibits mosquito fecundity and fertility

**Fig 1:**
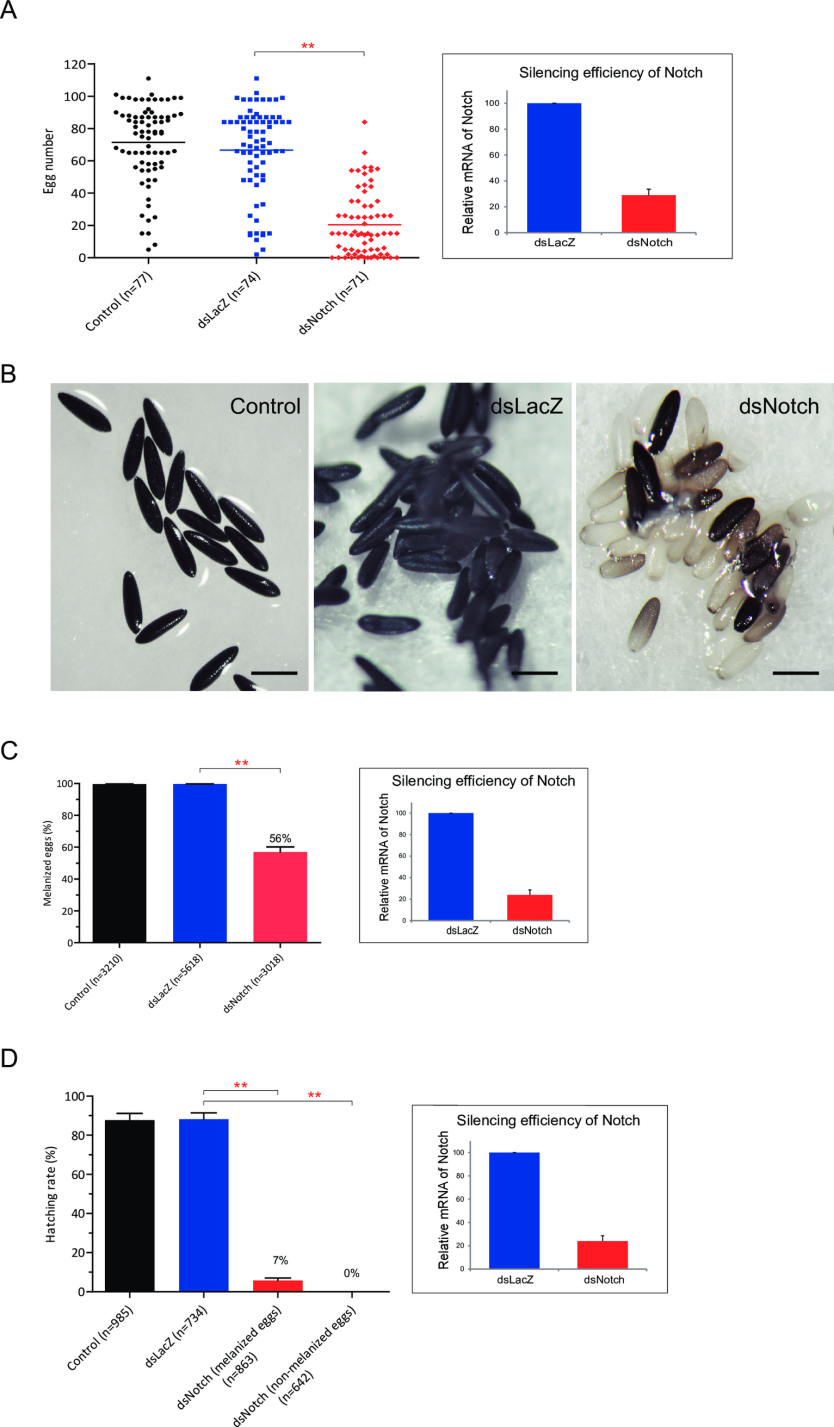
The effect of AaNotch on mosquito egg production, egg tanning and hatching. (A) The number of eggs per tube after egg induction relative to dsLacZ treatment by ANOVA (** = *p* < 0.001). The number in the parenthesis denotes the number of mosquitoes examined. (A, C, D) Silencing efficiency based on the levels of AaNotch transcript in female mosquitoes injected with dsLacZ and dsNotch, normalized against ribosomal protein s7 transcript (bar graph on right). (B, C) Oviposited eggs from control, dsLacZ-, and dsNotch-injected female mosquitoes. Eggs with hardened and darkened surface were counted as melanized eggs, whereas eggs that remained soft and white were counted as non-melanized eggs. (B) Scale bar = 0.5 mm. (C) The percentage of melanized eggs calculated from control, dsLacZ, or dsNotch-treated mosquitoes. At least three cohorts of mosquitoes were analyzed. (D) Eggs from control, dsLacZ, or dsNotch-treated mosquitoes subjected to deoxygenation-induced hatching. The numbers of the first instar larvae were counted. Numbers in the parentheses denotes total number of mosquitoes examined.

To investigate the role of AaNotch in mosquito fecundity, three-day-old mated female mosquitoes were injected with the dsRNA from LacZ or AaNotch and their egg productions were compared to female mosquitoes without any double-stranded RNA treatment. Egg production was examined four days PBM. Fig 1A showed that while AsNotch was efficiently knocked down (right panel), there was also a significant reduction in the number of eggs deposited by the AaNotch-silenced mosquitoes (20 ± 5) compared to that of controls (73 ± 6) or dsLacZ-treated (68 ± 5) mosquitoes (left panel). Follicles from dsRNA-treated mosquitoes randomly selected for stereomicroscopic observation showed no obvious difference in morphologies (S2A Fig) and the number of follicles between dsLacZ (90 ± 4) and dsNotch-treated (84 ± 5) mosquitoes, suggesting that silencing Notch does not affect follicle development. To elucidate the effect of AaNotch on egg tanning, we compared the percentages of melanized eggs relative to control, dsLacZ, and dsNotch-treated mosquitoes. A large portion (44%) of eggs from AaNotch-silenced mosquitoes remained soft and white at five days post-egg laying, while eggs from control and dsLacZ-treated mosquitoes were completely melanized (Fig 1B and 1C). Inset of Fig 1C showed that AsNotch was efficiently knocked down. A hatching assay was performed to determine the percentages of hatching larvae in control and dsLacZ- or dsNotch-treated mosquitoes. Although 100% of the eggs from control and dsLacZ-treated mosquitoes hatched, only 7% of the melanized eggs and none of the non-melanized eggs from AaNotch-silenced mosquitoes hatched (Fig 1D, right and left panel).

### The effect of AaNotch on the ultrastructure of mosquito eggs

**Fig 2:**
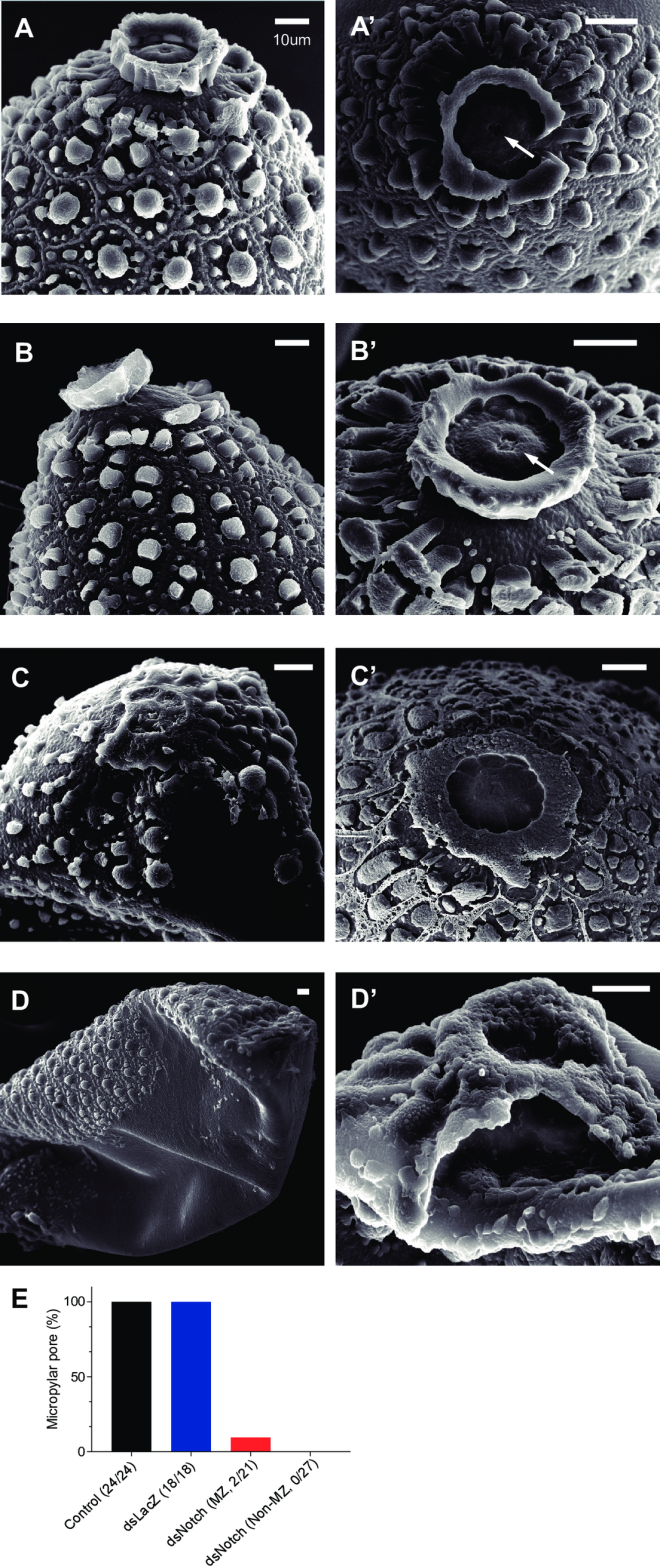
The effect of AaNotch on the ultrastructure of mosquito eggs. Mosquito eggs from control (A and A’), dsLacZ (B and B’), and dsNotch-treated (C, C’, D and D’) collected at 5 d after egg laying. Arrow: micropylar pore. Scale bar = 10 μm. (E) The percentage of eggs with complete micropylar pore formation. Number in parentheses denotes the number of eggs with complete micropylar pores divided by total number of eggs examined.

Our analysis of scanning electron microscopy on the ultrastructure of the eggs showed that while the micropyle and micropylar pores of eggs from control (Fig 2A and 2A’) and dsLacZ-treated (Fig 2B and 2B’) mosquitoes were detectable, those of both melanized (Fig 2C and 2C’) and non-melanized (Fig 2D and 2D’) eggs from AaNotch-silenced mosquitoes were missing. The ultrastructure of eggs from AaNotch-silenced mosquitoes indicates that these eggs would not be fertilized and hence, would have low fertility (Fig 1D). While micropylar pores could be detected in 100% of the eggs from control and dsLacZ-treated mosquitoes, they were present in only two of 21 melanized eggs from dsNotch-treated mosquitoes. Most importantly, none of the non-melanized eggs from dsNotch-treated mosquitoes exhibited micropylar pores (Fig 2E). (Eggs from control, dsLacZ- and dsNotch-treated female mosquitoes were treated with 50% bleach to remove the chorion and examined the interiors of these eggs.) We discovered that developing embryos were detected in eggs from control mosquitoes, but not in melanized or non-melanized eggs from dsNotch-treated mosquitoes (S3 Fig), showing that embryo development is impaired in the eggs of Notch-silenced mosquitoes. These results together strongly suggest that AaNotch is responsible for the formation of micropyle and hence, is crucial for mosquito fertility.

### Silencing AaNotch suppresses the expression of both sperm- and embryo-specific genes

**Fig 3:**
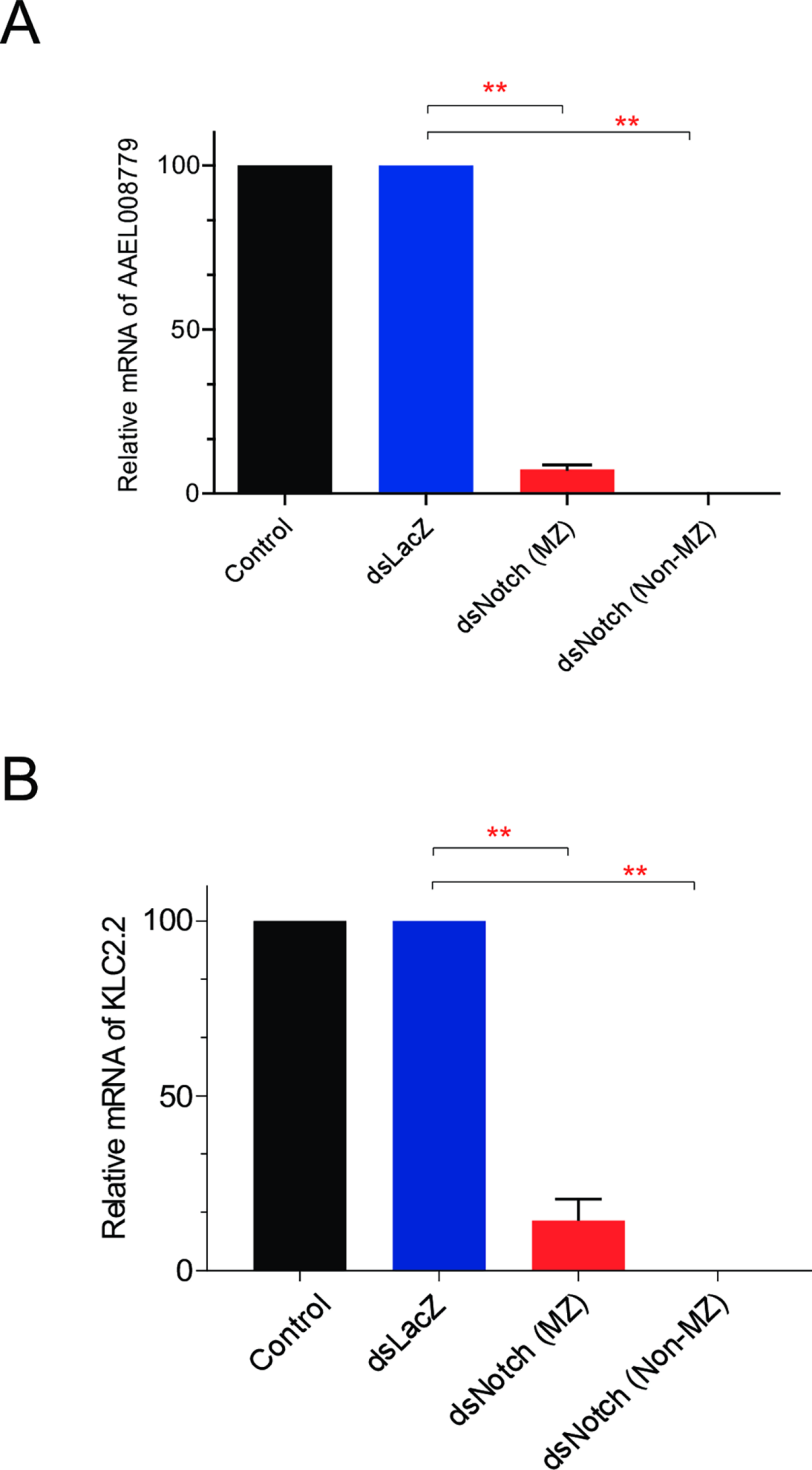
Quantitative PCR analysis of the expression of a sperm-specific and an embryo-specific gene. (A) Total RNA extracted from eggs of control, dsLacZ-, and dsNotch-treated mosquitoes, separated into melanized (MZ) and non-melanized (non-MZ) groups. (B) Total RNA of eggs from control and dsNotch-treated mosquitoes extracted at 5 h after egg-laying, representing melanized (MZ) and non-melanized (non-MZ) eggs. Data were analyzed with ANOVA (** = *p* < 0.001) comparing groups among brackets.

We hypothesized that fertility reduction in AaNotch-silenced mosquitoes results from the abolishment of micropyles. To examine the status of fertilization in non-melanized and melanized eggs from Notch-silenced mosquitoes, one sperm and one embryo-specific gene were selected as indicators to determine the status of fertilization and embryo development. Eggs from dsNotch-treated mosquitoes were then separated into melanized (dsNotch-MZ) and non-melanized (dsNotch-non-MZ) groups. The sperm-specific gene (Vector Base ID: AAEL008779) was detected in eggs from controls and dsLacZ-treated mosquitoes, but not in eggs from Notch-depleted mosquitoes, indicating that eggs from Notch-silenced mosquitoes had not been fertilized (Fig 3A). When we examined AaNotch embryonic development based on total RNA, we found that the early-embryo gene KLC2.2 was detected only in the eggs from controls and dsLacZ-treated mosquitoes, but not from Notch-silenced mosquitoes (Fig 3B). Taken together, these findings indicate that AaNotch controls the fertilization processes in female *A*. *aegypti*.

### Notch-dependent regulation of mosquito fertility is controlled by non-canonical Notch signaling pathway

When we analyzed effects of silencing the binding-ligand Delta and transcription factor CSL on Notch-controlled processes, we found that neither Delta not CSL affected mosquito fecundity (S4A Fig), egg melanization (S4B Fig), or fertility (S4C Fig). These results suggest that Notch-dependent regulation of mosquito fertility is likely not controlled by the canonical Notch signaling pathway.

**Fig 4:**
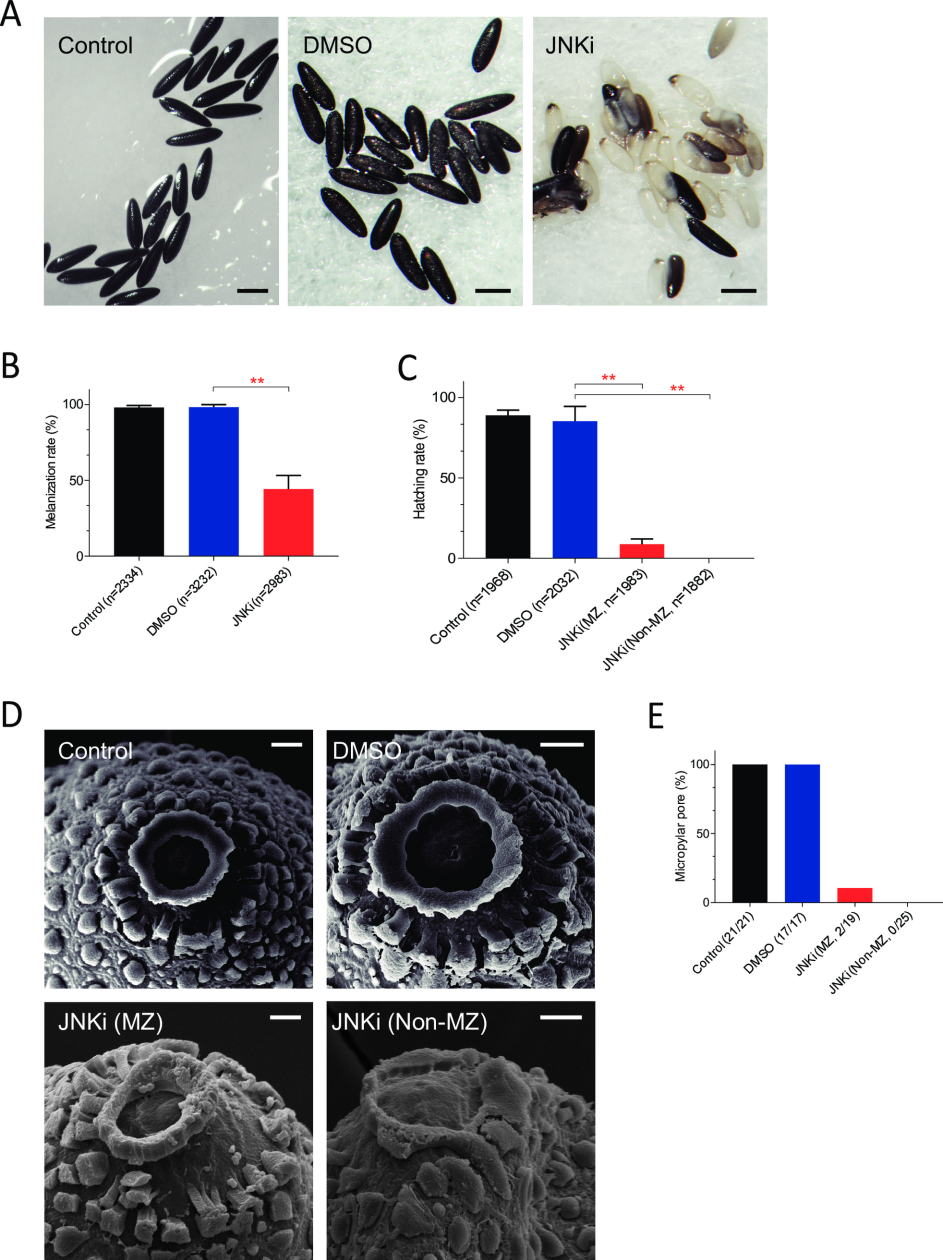
Chemical inhibition of JNK reduces egg tanning and hatching. (A) Total oviposited eggs from control, DMSO, or JNK inhibitor injected female mosquitoes (Scale bar = 0.5 mm.) (B) Melanized and non-melanized eggs (number in the parentheses denotes the total number of eggs counted). Y-axis is the percentage of melanized eggs of total eggs counted. (C) Eggs from control, DMSO, or JNK inhibitor (JNKi)-treated mosquitoes subjected to deoxygenation-induced hatching for melanized (MZ) and non-melanized (non-MZ) eggs. Number in the parentheses denotes total number of eggs examined. (D) Eggs from control, DMSO-, and JNK inhibitor-treated mosquitoes separated into melanized (MZ) and non-melanized (non-MZ) eggs. Scale bar = 10 μm. (E) Percentage of eggs with complete micropylar pore formation (Y-axis). Number in the parentheses denotes the number of eggs with complete micropylar formation divided by the total number counted.

JNK has been implicated as an important factor in egg-micropyle development in fruit flies and it participates in the CSL-independent, non-canonical Notch signaling pathway [25, 26]. It has been demonstrated that JNK phosphorylates transcription factors Jun and Fos, giving rise to a Jun/Fos dimer that activates transcription of target genes [29]. When we monitored the inhibition efficiency of the chemical inhibitor of JNK, we found that the expression of Jun was significantly inhibited with the treatment of SP600125 at dosages > 0.75 μg (S5A Fig). Furthermore, the specificity of SP600125 was confirmed because it did not affect the expression of *A*. *aegypti* p38 (AAEL008379) or EGFR (AAEL004391), both downstream components of other signaling pathways involving JNK (S5C and S5D Fig). However, inhibition of JNK phosphorylation in the non-canonical Notch pathway significantly reduced egg melanization (Fig 4A and 4B), fertility (Fig 4C), and micropyle formation (Fig 4D). Specifically, inhibition of JNK rendered 45% of eggs soft and white at five days after egg laying (Fig 4A and 4B). Our hatching assay showed that only 7% of the melanized eggs from mosquitoes treated with the JNK inhibitors hatched, while none of the non-melanized eggs hatched (Fig 4C). Ultrastructural analysis also revealed that eggs from mosquitoes treated with the JNK inhibitor had missing micropyles and micropylar pores (Fig 4D). Although micropylar pores could be detected in 100% of the eggs from control and DMSO-treated mosquitoes, the pores were present in only 2 of 19 (10%) of melanized eggs from mosquitoes treated with the JNK inhibitor (Fig 4E) and none of the non-melanized eggs had micropylar pores (Fig 4E). These results demonstrate that Notch-dependent regulation of mosquito fertility is controlled by a non-canonical Notch signaling pathway.

### Silencing AaNotch reduces JNK phosphorylation

**Fig 5:**
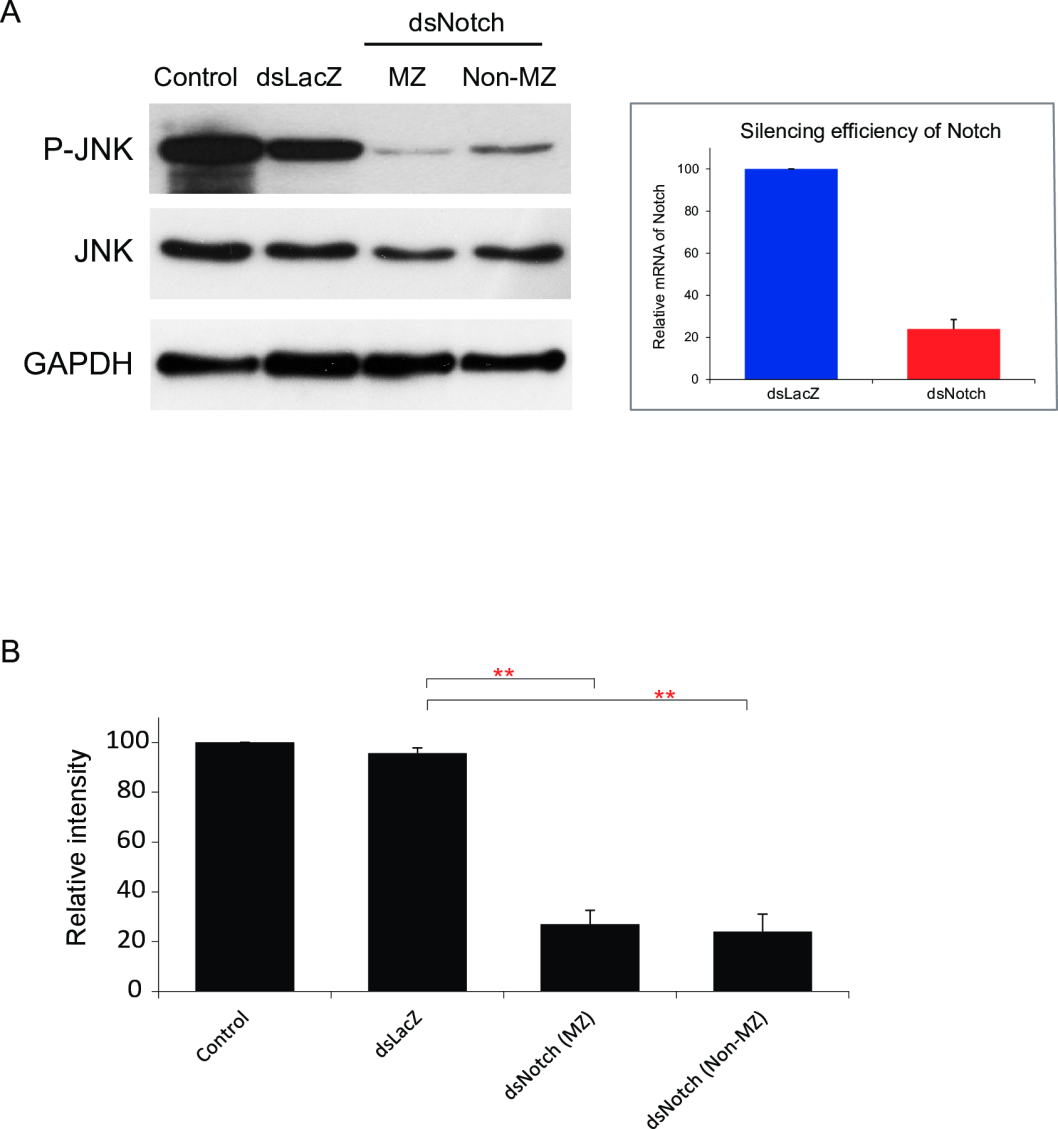
Silencing AaNotch reduces JNK phosphorylation. Total protein collected from control mosquitoes and mosquitoes treated with dsLacZ or dsNotch and controls 3 d post blood feeding for melanized (MZ) and non-melanized (non-MZ) eggs. Total JNK and GAPDH used as controls. Bar graph on the right shows RT-qPCR levels of AaNotch transcript in female mosquitoes injected with dsLacZ and dsNotch normalized against ribosomal gene s7. (B) The intensity of the mean of the signals relative to control condition (set at 100%). Bar graph shows the means of the p-JNK signal normalized against GAPDH.

Our results from three biological cohorts showed that silencing AsNotch significantly inhibited JNK phosphorylation in mosquitoes that produced either melanized eggs or non-melanized eggs (S6 Fig and Fig 5A, upper panel), while total JNK did not differ between controls, dsLacZ-, and dsNotch-treated mosquitoes (Fig 5A, middle and lower panel). When we examined signal intensities (quantified with Image J software) that had been normalized to controls, we found that there was a significant reduction in signal intensity of JNK phosphorylation in AaNotch-silenced mosquitoes producing either melanized eggs or non-melanized eggs (22% and 20%, respectively) (Fig 5B). Our results demonstrate that non-canonical Notch signaling is critical for the control of fertility in the mosquito *A*. *aegypti*.

## Discussion

The Notch pathway is an evolutionarily-conserved signaling pathway that functions during diverse developmental and physiological processes, including embryonic development, cell-fate specification, and stem cell maintenance [21–24]. One Notch receptor gene has been identified in *A*. *aegypti*, one in *Drosophila*, two in *C*. *elegans* (*Lin-12* and *Glp-1*) and four in mammals (*Notch1*, *Notch2*, *Notch3*, and *Notch4*) [30–34]. The initial study of Notch was on a mild phenotype at the wing tip of *Drosophila* [18]. Notch study has since grown into an interdisciplinary field involving genetics, developmental, cellular, and molecular biology. However, the function of the Notch signaling pathway in *A*. *aegypti* still remains largely unknown.

In this paper, we demonstrate the crucial role of non-canonical Notch signaling in the control of a sterile-like phenotype in the mosquito *A*. *aegypti* and indicate that AaNotch plays an important role in regulating mosquito fecundity and fertility. Notch signaling has been demonstrated to be involved in the regulation of oogenesis in *Drosophila* [22–24] by regulating multiple aspects of somatic follicle cell differentiation in *Drosophila* ovaries, including differentiation of stalk and polar cells [35, 36]. In addition, Notch signaling (in concert with an ecdysone receptor) has been found to control of dorsal volume of appendage tubes by promoting apical re-expansion and lateral shortening of dorsal appendage-forming follicle cells [35]. Thus, Notch signaling differs in its physiological functions between mosquitoes and *Drosophila*. AaNotch also affects melanization of mosquito eggs, but the mechanism for how this occurs is not known and so must be investigated further.

Our ultrastructural analysis showed that micropyle and micropylar pores do not occur in eggs from AaNotch-silenced mosquitoes. Because mosquito sperms penetrate eggs through micropylar pores, fertilization cannot occur without an intact micropylar pore. Our results suggest that AaNotch signaling modifies micropylar pore formation in mosquito eggs, essentially resulting in defective fertilization.

By silencing Delta (ligand of Notch) and CSL (transcription factor of Notch signaling), we discovered that Notch-dependent regulation of reproduction is not controlled by canonical Notch signaling pathway, but rather that there might be a non-canonical Notch signaling role in the regulation of mosquito fecundity and fertility. Zecchini et al. showed that, in *Drosophila*, Notch plays a role in the patterning of dorsal epidermis through a JNK-signaling pathway [25]. The JNK cascade is essential for the correct morphogenesis of dorsal appendages and micropyle formation during *Drosophila* oogenesis [25]. We found that treating mosquitoes with JNK inhibitor significantly reduced micropylar pore formation, egg melanization, and hatching. These results indicate that JNK is essential for controlling fertility in mosquitoes. We also showed that silencing AsNotch inhibits JNK phosphorylation, thus suggesting that AaNotch controls an upstream process of JNK phosphorylation and so controls mosquito reproduction through a non-canonical Notch signaling pathway. A recent report indicated that Notch signaling controls gut actin cytoskeleton in mosquitoes via micro-RNA 275 [37]. Thus, it is very likely that Notch signaling controls multiple physiological functions in mosquitoes.

In recent years, use of the mosquito endosymbiont bacterium *Wolbachia pipientis* has become a promising strategy for controlling mosquitoes that carry diseases [38, 39]. *Wolbachia* is well known for its ability to induce cytoplasmic incompatibility, which causes reproductive abnormalities in its insect host. A *Wolbachia*-based strategy has been used in several field study sites to control dengue [38, 39]. An alternative, transgenic-based strategy has also been developed to sterilize insect vectors to reduce their populations [40, 41]. Both of these strategies aim to reduce vector populations by controlling mosquito reproduction. In our study, we discovered that the non-canonical Notch signaling pathway controls *A*. *aegypti* reproduction. Therefore, it is possible that modulating AaNotch could be used as an alternative strategy for controlling *A*. *aegypti* populations.

In summary, our study identified a fundamental role of non-canonical Notch signaling pathway in the regulation of mosquito fertility. Because mosquito-borne diseases remain an important threat to several billion people worldwide who inhabit tropical and subtropical countries, novel or alternative approaches for vector control are urgently needed. *Wolbachia*-based elimination strategies [42, 43] and engineered genetic approaches for vector control [40, 41] shed new light on the control of mosquito-borne diseases. Our study reveals the pleiotropic action of non-canonical Notch signaling in the control of mosquito reproduction, thereby providing new insight for developing environmentally-friendly methods to target vector reproduction.

## Supporting information

S1 Fig The expressional pattern of Notch in mosquitoes. Total RNA of various tissues (FB: fat body, MG: midgut, OV: ovary and CC: carcass) from female mosquitoes collected during the pre-vitellogenic stage (PV) PBM. Asterisks indicate statistical significance of ANOVA (*** = *p* < 0.001) at 24 h PBM to that of PV stage mosquitoes.
(TIF)

S2 Fig Status of follicle cells in dsLacZ and dsNotch-treated mosquitoes. (A) Ovaries from dsLacZ and dsNotch-treated mosquitoes collected at various time periods after eclosion (not fed blood). (B) The numbers of follicle cells in ovaries from dsLacZ and dsNotch-treated mosquitoes calculated at 72 h PBM.
(TIF)

S3 Fig Development of embryo from dsNotch-treated mosquitoes was abolished. Eggs from control and dsNotch-treated mosquitoes collected 5 d after egg deposition for melanized (MZ) and non-melanized (non-MZ) eggs. Scale bar = 0.5 mm.
(TIF)

S4 Fig The effect of AaDelta on mosquito egg production, egg tanning and hatching. (A) The number of eggs in each tube was counted 4 days after egg induction. Number in the parentheses denotes the total number of mosquitoes examined. (B) The percentage of melanized eggs from the control, dsLacZ, dsNotch, dsDelta, and dsCSL-treated mosquitoes. (C) Eggs from control, dsLacZ, dsNotch, dsDelta and dsCSL-treated mosquitoes subjected to deoxygenation-induced hatching. The number of the first instar larvae was counted. (D) RT-PCR analyzes of the mRNA level of Notch, Delta, and CSL in female mosquitoes injected with dsLacZ, dsNotch, dsDelta, or dsCSL.
(TIF)

S5 Fig Inhibition efficiency of JNK inhibitor. (A) Total RNA collected 3 d after treatment, with the expression of Jun in un-manipulated control mosquitoes set at 100%. (B, C, D) Number of melanized (MZ) and non-melanized (non-MZ) eggs). Expressions quantified by qPCR and normalized against ribosomal gene s7, wherein the expression of Jun in un-manipulated control mosquitoes was set at 100%: (B) Jun, (C) *Aedes aegypti* p38 (Vector Base ID: AAEL008379), and (D) *A*. *aegypti* EGFR (Vector Base ID: AAEL004391).
(TIF)

S6 Fig Original blots for Fig 5A. Results of three separate Western blot experiments: (A) JNK phosphorylation analyzed with anti-phospho-JNK antibody (Promega, V7931), (B) with anti-JNK (Santa Cruz sc-571), and (C) with anti-GAPDH (GeneTex, GTX100118) antibodies.
(TIF)

S1 Table Gene accession numbers and primers used in this study. (PDF)
