# Supplementary figures and images for: The non-canonical Notch signaling is essential for the control of fertility in Aedes aegypti
Source: PLoS Negl Trop Dis. 2018 Mar 5;12(3):e0006307. doi: 10.1371/journal.pntd.0006307 (PMC5854436; doi:10.1371/journal.pntd.0006307)

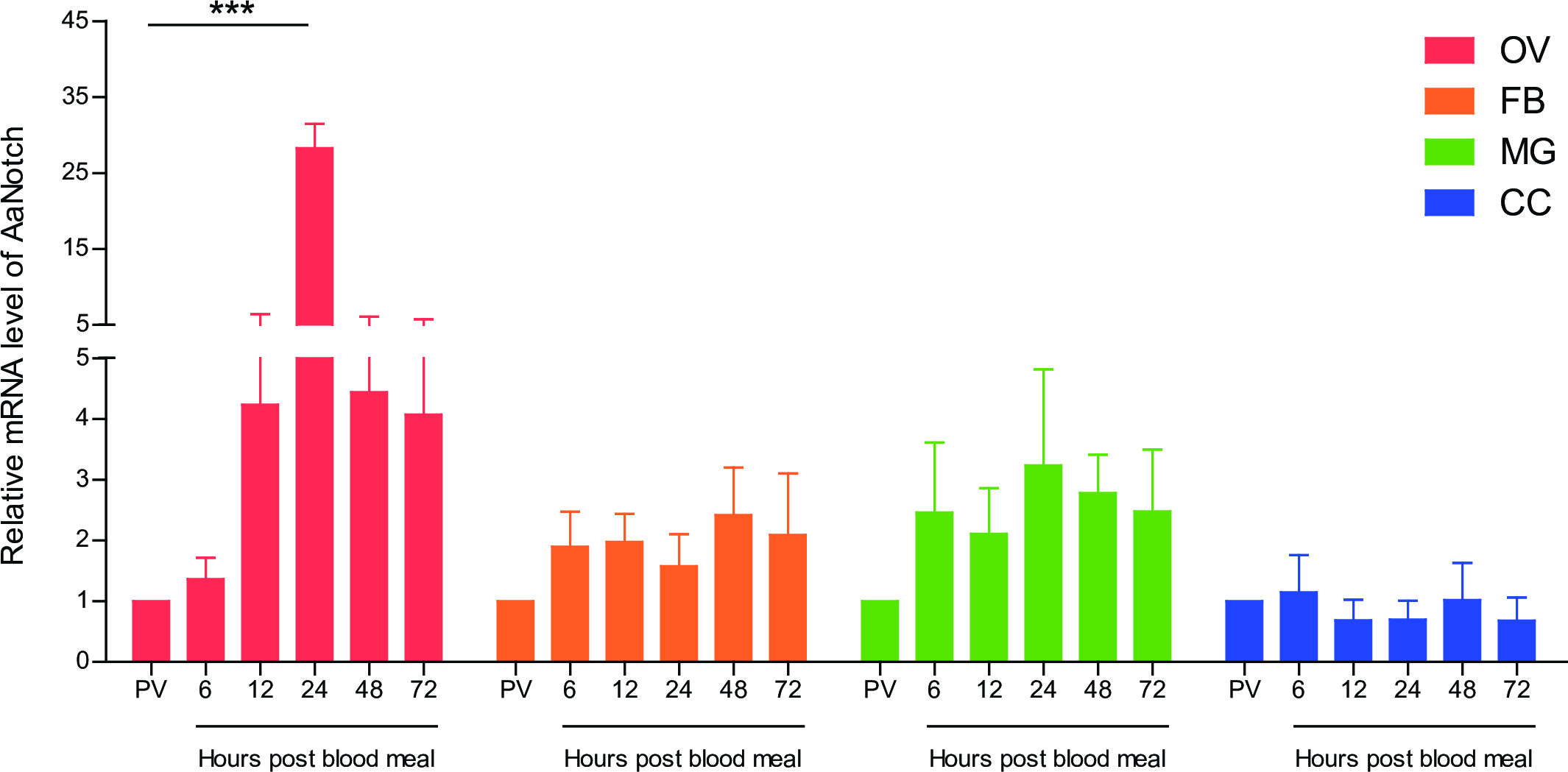

Supplement: S1 Fig — Total RNA of various tissues (FB: fat body, MG: midgut, OV: ovary and CC: carcass) from female mosquitoes collected during the pre-vitellogenic stage (PV) PBM. Asterisks indicate statistical significance of ANOVA (*** = p < 0.001) at 24 h PBM to that of PV stage mosquitoes. (TIF) [file pntd.0006307.s001.tif]

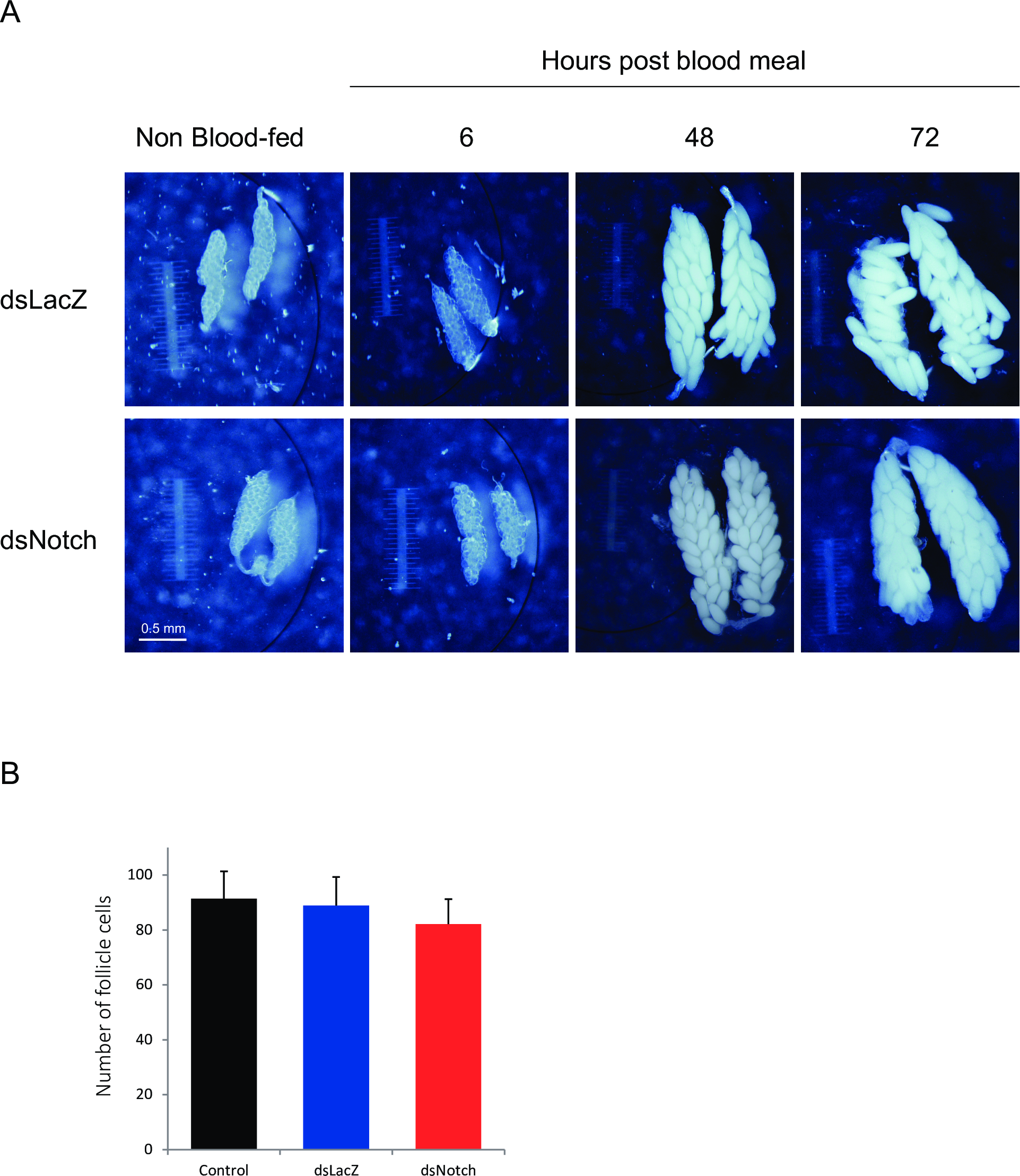

Supplement: S2 Fig — (A) Ovaries from dsLacZ and dsNotch-treated mosquitoes collected at various time periods after eclosion (not fed blood). (B) The numbers of follicle cells in ovaries from dsLacZ and dsNotch-treated mosquitoes calculated at 72 h PBM. (TIF) [file pntd.0006307.s002.tif]

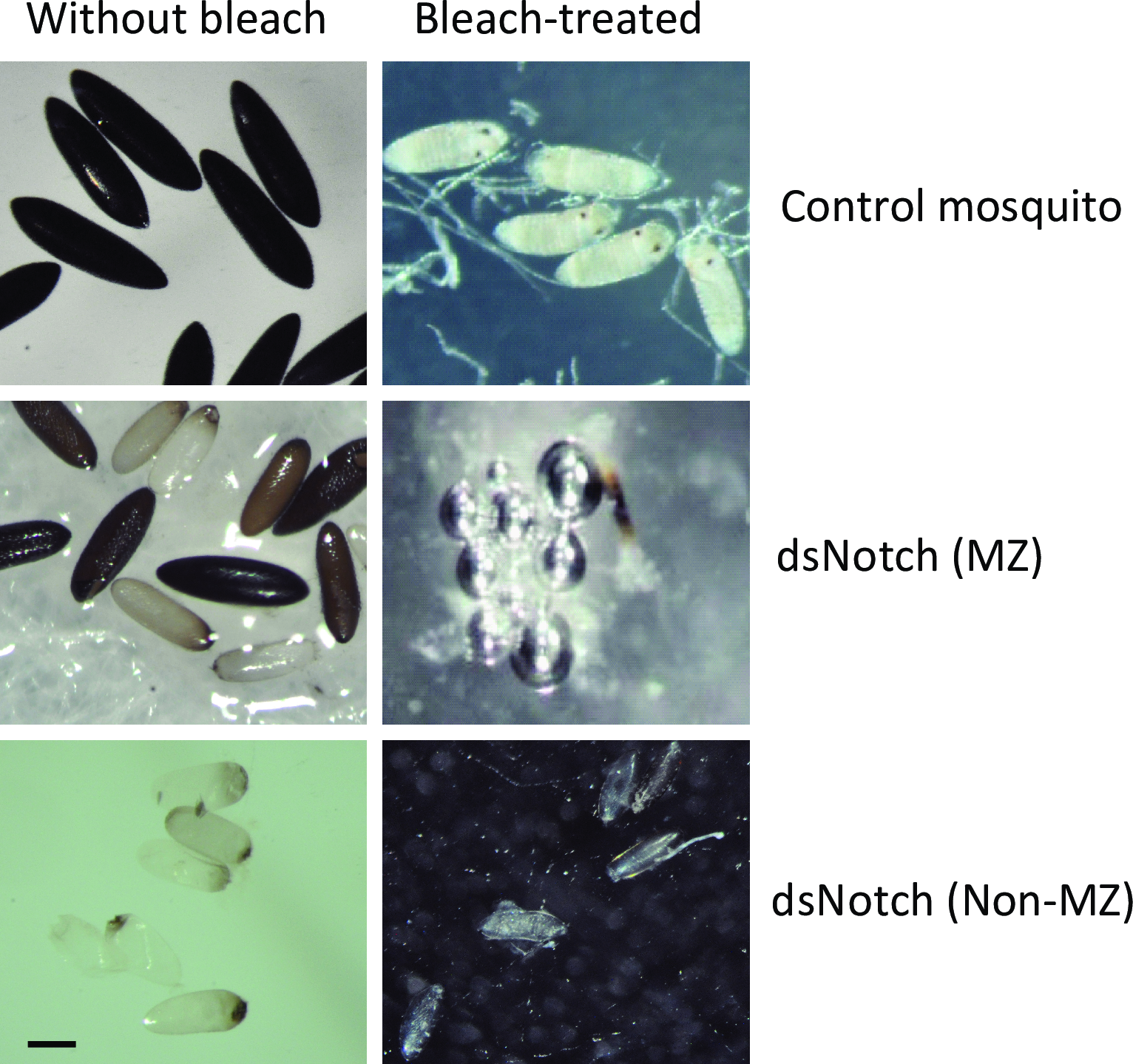

Supplement: S3 Fig — Eggs from control and dsNotch-treated mosquitoes collected 5 d after egg deposition for melanized (MZ) and non-melanized (non-MZ) eggs. Scale bar = 0.5 mm. (TIF) [file pntd.0006307.s003.tif]

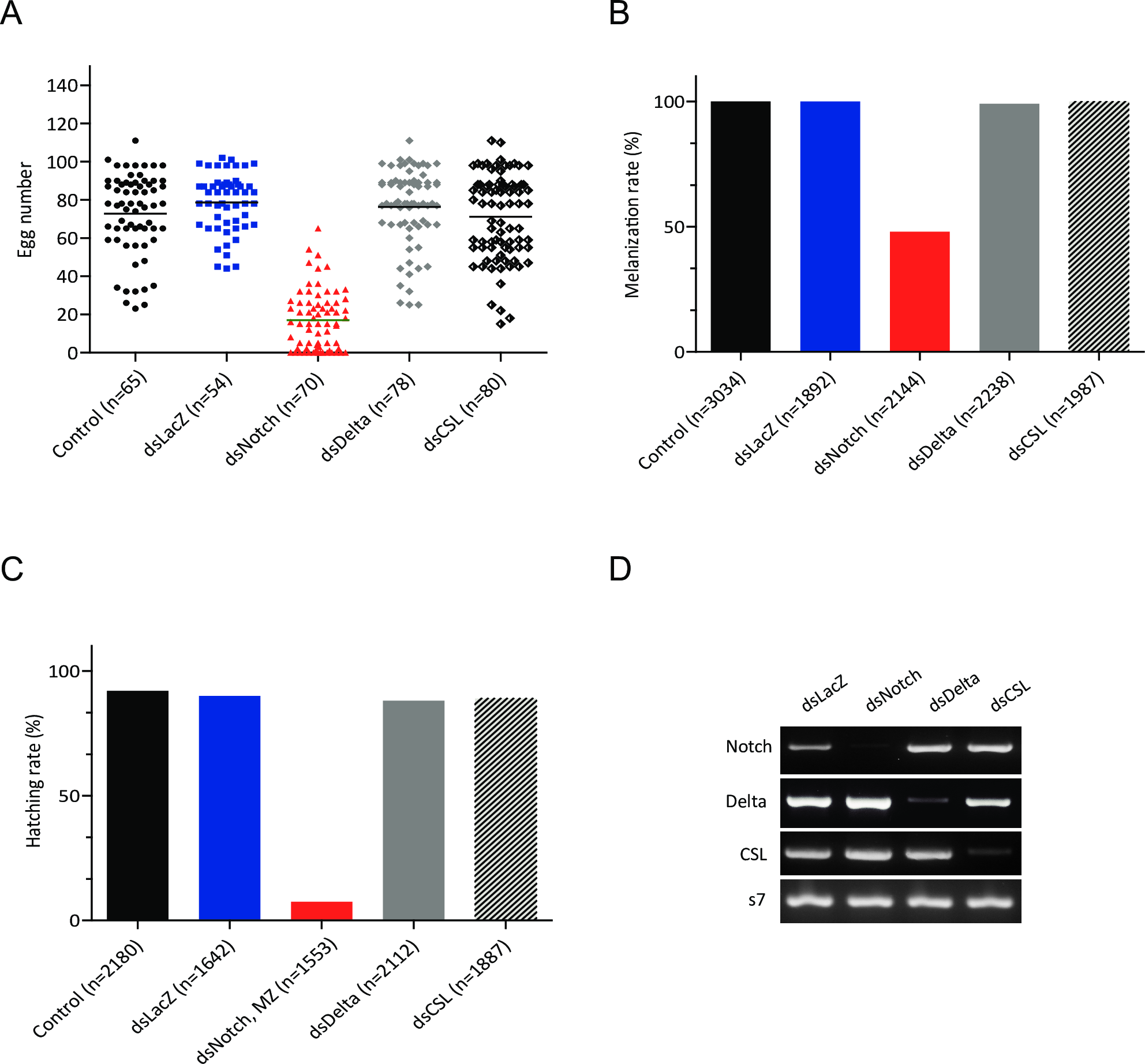

Supplement: S4 Fig — (A) The number of eggs in each tube was counted 4 days after egg induction. Number in the parentheses denotes the total number of mosquitoes examined. (B) The percentage of melanized eggs from the control, dsLacZ, dsNotch, dsDelta, and dsCSL-treated mosquitoes. (C) Eggs from control, dsLacZ, dsNotch, dsDelta and dsCSL-treated mosquitoes subjected to deoxygenation-induced hatching. The number of the first instar larvae was counted. (D) RT-PCR analyzes of the mRNA level of Notch, Delta, and CSL in female mosquitoes injected with dsLacZ, dsNotch, dsDelta, or dsCSL. (TIF) [file pntd.0006307.s004.tif]

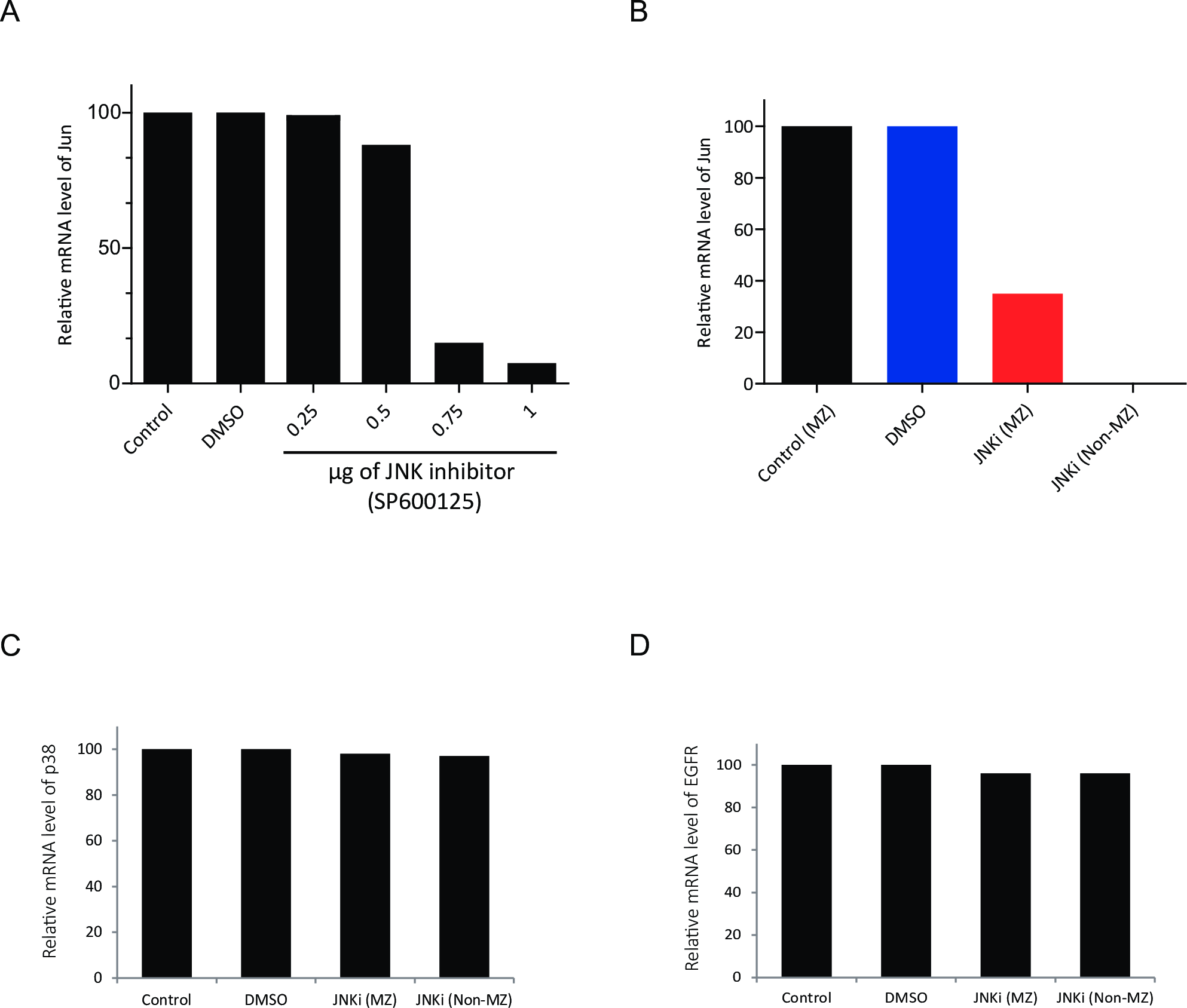

Supplement: S5 Fig — (A) Total RNA collected 3 d after treatment, with the expression of Jun in un-manipulated control mosquitoes set at 100%. (B, C, D) Number of melanized (MZ) and non-melanized (non-MZ) eggs). Expressions quantified by qPCR and normalized against ribosomal gene s7, wherein the expression of Jun in un-manipulated control mosquitoes was set at 100%: (B) Jun, (C) Aedes aegypti p38 (Vector Base ID: AAEL008379), and (D) A. aegypti EGFR (Vector Base ID: AAEL004391). (TIF) [file pntd.0006307.s005.tif]

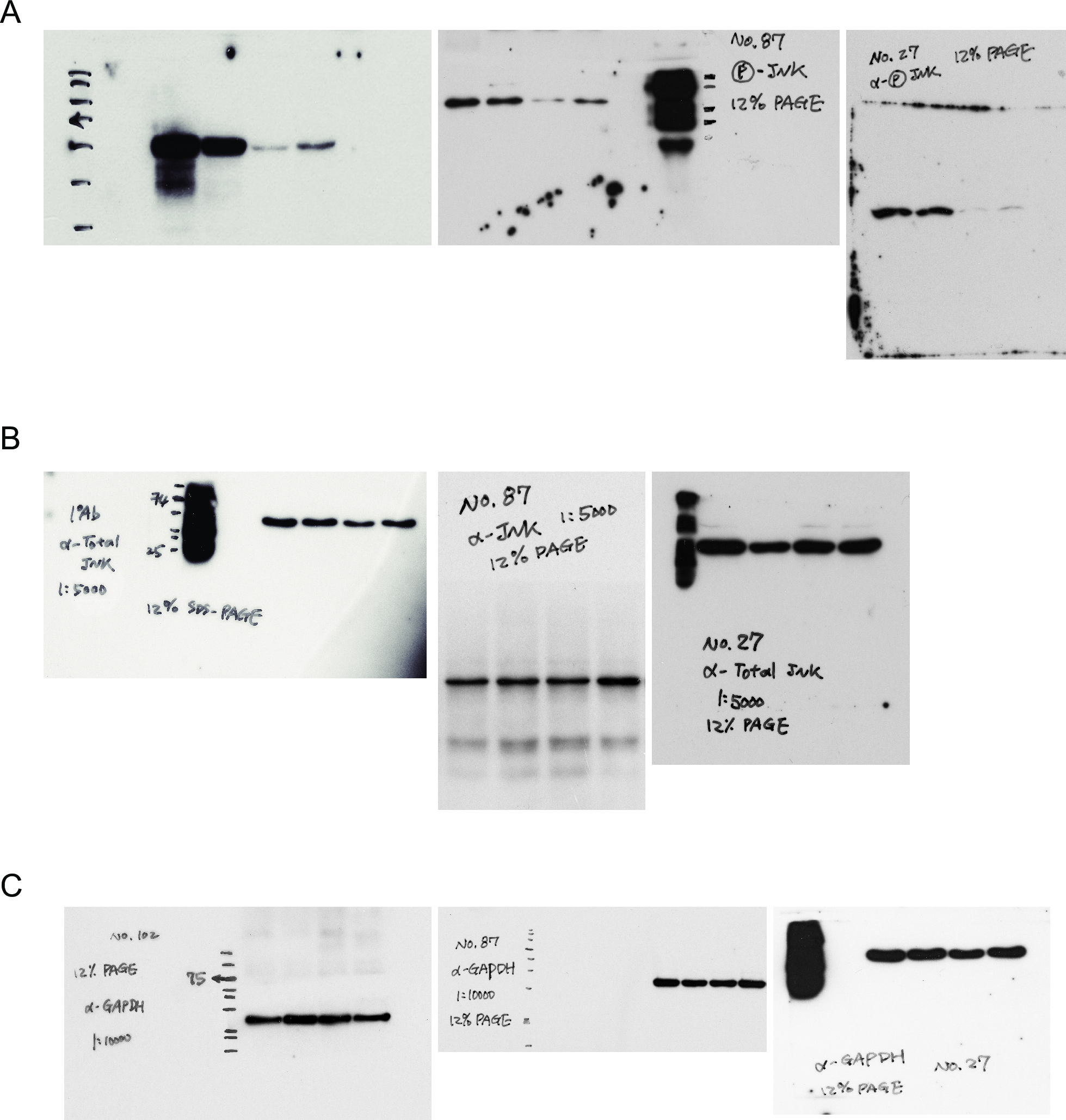

Supplement: S6 Fig — Results of three separate Western blot experiments: (A) JNK phosphorylation analyzed with anti-phospho-JNK antibody (Promega, V7931), (B) with anti-JNK (Santa Cruz sc-571), and (C) with anti-GAPDH (GeneTex, GTX100118) antibodies. (TIF) [file pntd.0006307.s006.tif]
