# Supplementary material for: The non-canonical Notch signaling is essential for the control of fertility in Aedes aegypti
Source: PLoS Negl Trop Dis. 2018 Mar 5;12(3):e0006307. doi: 10.1371/journal.pntd.0006307 (PMC5854436; doi:10.1371/journal.pntd.0006307)
Supplement: S1 Table — (PDF) [file pntd.0006307.s007.pdf]

S1 Table. Gene accession numbers and primers used in this study

Primer	Vector Base ID	Forward sequence (5' → 3')	Reverse sequence (5' → 3')	Annealing temp. (℃)	Size (bps)
Notch RNAi	AAEL001210	TAATACGACTCACTATAGGGACGTGGTTCCCAGAAGGATT	TAATACGACTCACTATAGGGCCCAGTCTTATCCATGGTCG	60.5	528
Notch qPCR	AAEL001210	ATCGGGATGCTCAAGATGAC	TGATCATCCGAAATCTGCAC	51	466
Delta RNAi	AAEL011396	TAATACGACTCACTATAGGGGGATATAGCGGTACTCGATG	TAATACTACTCACTATAGGGGTCATCTTTCTCCTGCTCAC	60.5	591
Delta qPCR	AAEL011396	ACTACCTCACGATCACCAGA	GGATAGGGTTCACACTCGT	51	366
CSL RNAi	AAEL006419	TAATACGACTCACTATAGGGACGAGAGCGAGTCAGAGGAG	TAATACGACTCACTATAGGGCGTGAAAGTTAGCCCCGTAG	60.5	647
CSL CHK	AAEL006419	CCACCCAACTATGTGCCTTT	ACATCCGTTCGGTGTCTTTC	50	606
SPM779 qPCR	AAEL008779	TCCTTGTGGACCCTGTGTAA	AATCTGTGGCTTCAGTTGG	53	499
KLC2.2 qPCR	AAEL021010	CCCGAAGCCATTAAACAC	TGATTCACTGTCACCATTCTGTAC	55	465
S7 qPCR	AAEL009496	GAAAAGATGGCATGGGAAGTG	CTCCTGCTTCCGTTTGATTACC	52	69
Jun qPCR	AAEL003505	CTCCATGCGCGAAAAAGATA	TGTGTAGTTCGTTGTTCGT	54	261
P38 qPCR	AAEL008379	CCCAGTCCTTAAAGGCACG	CGCTTAAGGCCCTTAGAGATT	56	267
EGFR qPCR	AAEL004391	ACCCTTTGGATATACCCGCG	GCCTATTGGCCCGTGTGGGAAT	56	289
